# Reversal of trends in global fine particulate matter air pollution

**DOI:** 10.1038/s41467-023-41086-z

**Published:** 2023-09-02

**Authors:** Chi Li, Aaron van Donkelaar, Melanie S. Hammer, Erin E. McDuffie, Richard T. Burnett, Joseph V. Spadaro, Deepangsu Chatterjee, Aaron J. Cohen, Joshua S. Apte, Veronica A. Southerland, Susan C. Anenberg, Michael Brauer, Randall V. Martin

**Affiliations:** 1 Department of Energy, Environmental & Chemical Engineering, Washington University in St. Louis, St. Louis, MO USA; 2 Institute for Health Metrics and Evaluation, University of Washington, Seattle, WA USA; 3 Population Studies Division, Health Canada, Ottawa, ON Canada; 4 Spadaro Environmental Research Consultants (SERC), Philadelphia, PA USA; 5 European Centre for Environment and Health, World Health Organization (Consultant), Bonn, North Rhine-Westphalia Germany; 6 Health Effects Institute, Boston, MA USA; 7 Department of Civil and Environmental Engineering, University of California, Berkeley, CA USA; 8 School of Public Health, University of California, Berkeley, Berkeley, CA USA; 9 Milken Institute School of Public Health, George Washington University, Washington, DC USA; 10 School of Population and Public Health, University of British Columbia, Vancouver, BC Canada; 11 Present Address: Office of Atmospheric Protection, Climate Change Division, U.S. Environmental Protection Agency, Washington, D.C. USA

**Keywords:** Environmental impact, Atmospheric chemistry

## Abstract

Ambient fine particulate matter (PM_2.5_) is the world’s leading environmental health risk factor. Quantification is needed of regional contributions to changes in global PM_2.5_ exposure. Here we interpret satellite-derived PM_2.5_ estimates over 1998-2019 and find a reversal of previous growth in global PM_2.5_ air pollution, which is quantitatively attributed to contributions from 13 regions. Global population-weighted (PW) PM_2.5_ exposure, related to both pollution levels and population size, increased from 1998 (28.3 μg/m^3^) to a peak in 2011 (38.9 μg/m^3^) and decreased steadily afterwards (34.7 μg/m^3^ in 2019). Post-2011 change was related to exposure reduction in China and slowed exposure growth in other regions (especially South Asia, the Middle East and Africa). The post-2011 exposure reduction contributes to stagnation of growth in global PM_2.5_-attributable mortality and increasing health benefits per µg/m^3^ marginal reduction in exposure, implying increasing urgency and benefits of PM_2.5_ mitigation with aging population and cleaner air.

## Abstract (web summary)

Global fine particulate matter air pollution recently pivots from increase to decrease as inferred from satellite observations, driven by unprecedented exposure reduction in China and slowed exposure growth in South Asia, the Middle East and Africa.

## Introduction

Global ambient fine particulate matter (PM_2.5_) air pollution is responsible for millions of annual premature deaths^1–5^, ~1 year of reduced life expectancy^6^, and trillions of US dollars of social costs^7–9^. Global population exposure to PM_2.5_ is skewed by the broad co-existence of high population density and high PM_2.5_ concentration^10–12^, especially in South and East Asia. Nearly half (47%) of current global PM_2.5_-attributable deaths are due to traditional sources of air pollution exposure such as fossil and solid biofuel combustion^13^, which can be mitigated. High-income countries in Europe and North America have for decades regulated emissions from major air pollution sources^14–16^. Ambient PM_2.5_ levels across the US and Canada, for example, have decreased by 64% from 1981 to 2016^17^. Meanwhile PM_2.5_ exposure deteriorated in Asia following rapid industrialization and urbanization, as inferred mainly by satellite observations^18–22^. Both the increasing PM_2.5_ exposure and rapid population growth in Asia have led to a steady increase of the global PM_2.5_-associated health burden over at least 1990-2010^3,23,24^.

Since the early 2010s, China implemented increasingly rigorous measures to alleviate its severe air pollution^25–27^, resulting in substantial population-weighted (PW) PM_2.5_ reductions (in excess of 15 μg/m^3^ within 8 years) as determined by nationwide ground monitoring, satellite-derived estimates and air quality modeling^25,28,29^. The consequent national health benefits (e.g., a reduction of ~0.4 million attributable annual deaths) are substantial^25,26^. Additionally, evidence is emerging that the growth rate of the PM_2.5_ and total aerosol burden is slowing in other key regions including India^23,28,30^, North Africa^31,32^, the Middle East^32–34^, Central Africa^31^, and the Amazon^35,36^. There is need to comprehensively interpret, within a global context, how these regional changes affect global PM_2.5_ air pollution and its health impacts.

Here, we quantify how PW PM_2.5_ air pollution and health burdens have changed during 1998-2019, both globally and in 13 regions, with a quantification of regional contribution to changes in global exposure. We use a combination of high-resolution (0.01°, approximately 1 km^2^) satellite-derived global PM_2.5_ data, territory-, age- and disease-specific mortality data, and a concentration-response model linking PM_2.5_ outdoor concentrations to adverse health impacts. We present a comprehensive analysis revealing a timely reversal of global trends in PW PM_2.5_, largely driven by reductions in China and bolstered by mitigation efforts across different regions. The declining trend in recent years points toward a possible path of continuing and sustained improvements in global air quality and reductions in the associated health burdens.

## Results and discussion

### Reversal in global population-weighted PM_2.5_ trends

**Fig. 1:**
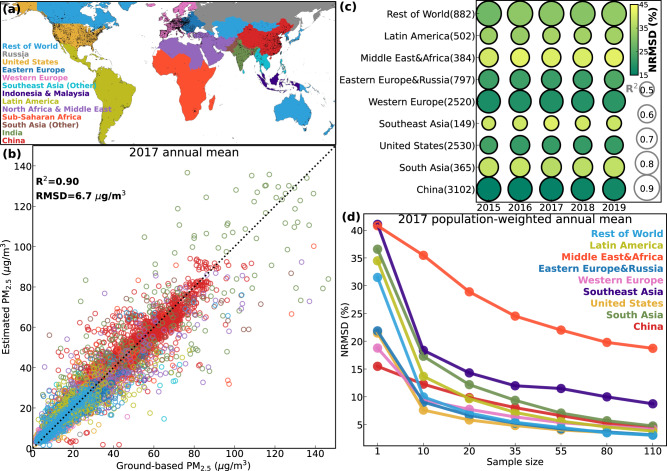
Validation of estimated PM_2.5_. **a** Definition of 13 regions (consistent color-coding throughout the paper) and the distribution of monitoring sites (black dots). **b** Scatter plot (color-coded for the 13 regions) of annual mean PM_2.5_ from ground-based monitors (*X*-axis) and the 1 km satellite-drived estimates (*Y*-axis) in 2017, with overall statistical metrics indicated at the upper-left. **c** Regional distribution of *R*^2^ (size) and normalized root mean square difference (NRMSD, color, Eqs. 1, 2) for the annual mean PM_2.5_ through 2015-2019. Number of available sites for evaluation are indicated in the brackets following the region names. **d** Bootstrapped NRMSD of regional population-weighted PM_2.5_ as a function of sample size in 2017. The map is created using packages cartopy^56^ and matplotlib^57^ in Python.

We use recently updated monthly global PM_2.5_ data gridded at ~1 km^2^ resolution over 1998-2019, as derived from satellite observations of aerosol optical depth, chemical transport modeling, and ground monitoring data^28^ (“Methods” section). Annual mean estimates are consistent (e.g., *R*^2^ = 0.90 in 2017) with ground-based observations (Fig. 1 and Supplementary Section S1).

**Fig. 2:**
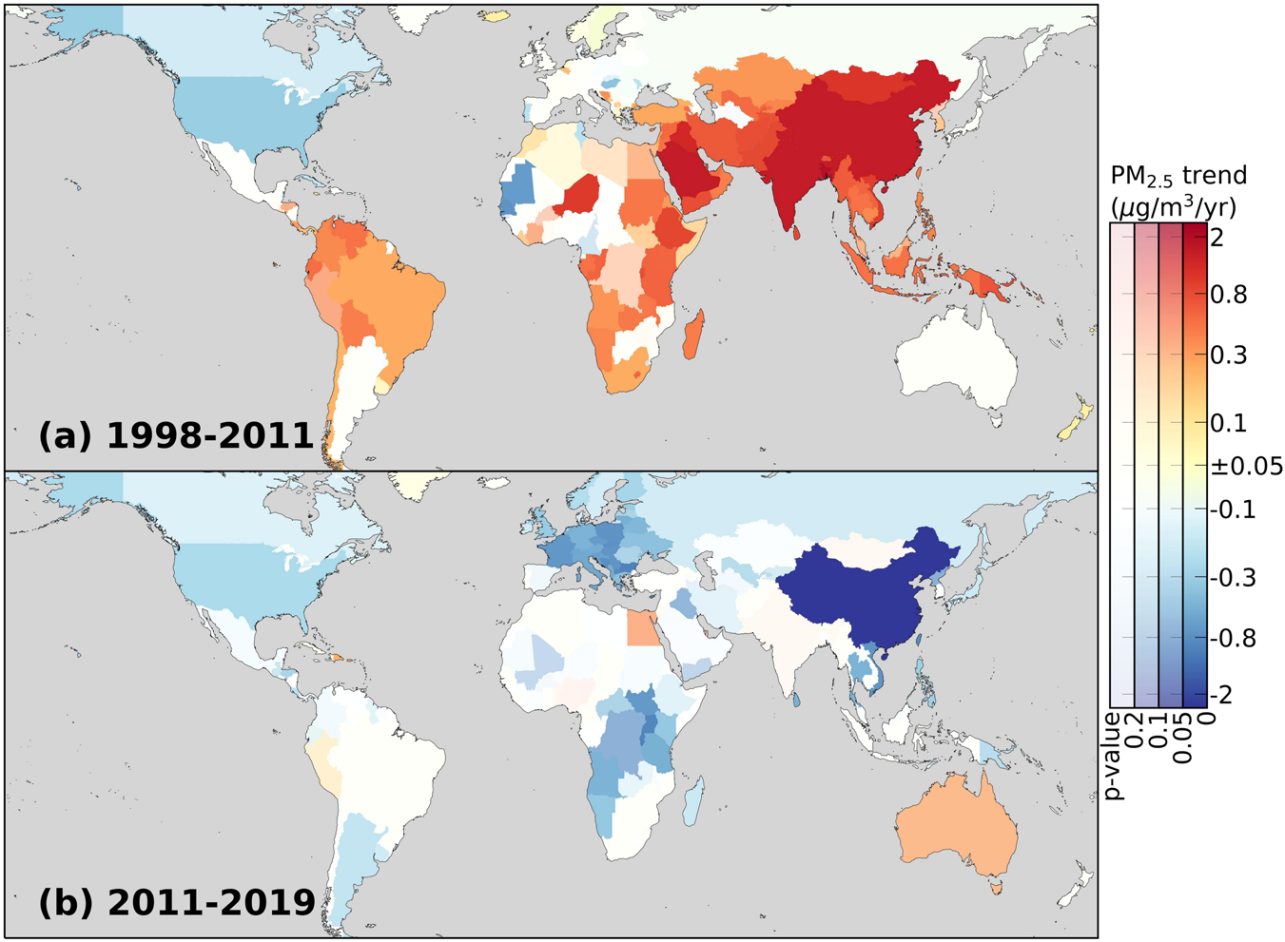
General reversal of trends in PM_2.5_ air pollution around the world. Maps show trends in population-weighted PM_2.5_ for 204 territories over (**a**) 1998-2011 and (**b**) 2011-2019. Trends with lower level of statistical significance (i.e., higher *p*-values) are plotted with more transparent colors. The maps are created using packages cartopy^56^ and matplotlib^57^ in Python.

Annual mean PW PM_2.5_ exposure changed substantially across 204 global territories (defined by the World Health Organization) over 1998–2019. Before 2011 (Fig. 2a), significant (*p* < 0.05) growth in PM_2.5_ exposure occurred widely across 87 territories (population 4.4 billion) mainly in Asia, Africa and South America, while only 15 territories (population 0.4 billion) exhibited significant reductions. Instead, during 2011–2019 (Fig. 2b), the breadth of areas with significant decreases expanded substantially (58 territories, population 3.0 billion) to include Europe, Asia Pacific, Southeast Asia, Russia, Sub-Saharan Africa, and most notably, China. Other regions whose PW PM_2.5_ levels were clearly increasing before 2011, including South Asia, North Africa, and South America, also exhibited decreases in the upward trends. Only five territories (in the Caribbean and Pacific, population 14.3 million) exhibited significant positive trends during 2011–2019. Overall, at the global level there has been a reversal in the pre-2011 increase of PW PM_2.5_. The regional trends are largely connected with local air quality management policies in each region (Supplementary Section S2).

**Fig. 3:**
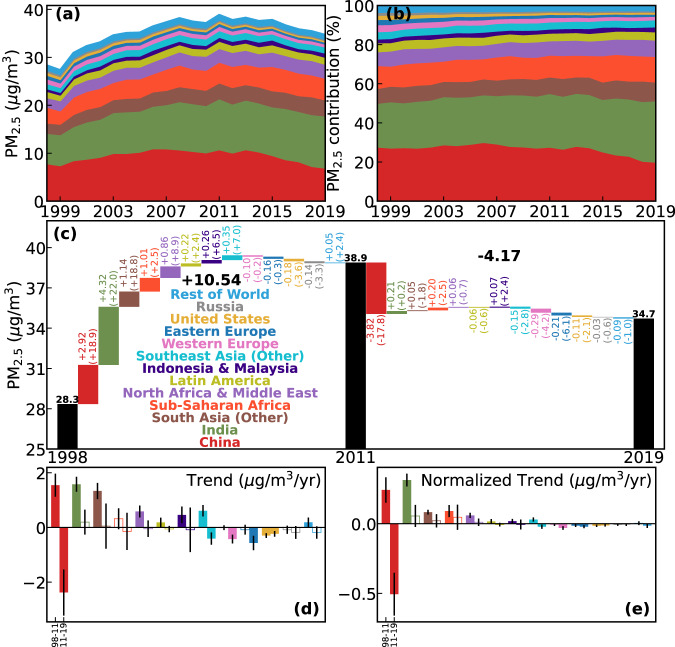
Reversal of global PM_2.5_ exposure growth dominated by China. Time series of absolute (**a**) and relative share (**b**) of global population-weighted (PW) PM_2.5_ concentrations in 13 regions (colored) from 1998 to 2019. **c** Contribution of each region to the changes in global PW PM_2.5_ among 1998, 2011, and 2019. The two large black numbers define the total differences from global exposures indicated by black bars (and numbers above them), and the colored bars and numbers attribute the differences into regional contributions (numbers in bracket indicates the absolute change before normalized to global population). Trends (filled bars indicate significant trends with *p*-values < 0.05 and empty bars indicate insignificant trends) in population-weighted PM_2.5_ before (**d**) and after (**e**) normalization to global population over each region for 1998-2011 (i.e., the left bar for each colored region) and 2011–2019 (right). Error bars in **d** and **e** indicate the 95% confidence intervals of the trends.

Consequently, global annual mean PW PM_2.5_ peaked in 2011 (38.9 μg/m^3^, Fig. 3a), with trends ±95% confidence intervals (CI) of 0.8 ± 0.2 μg/m^3^/year during 1998–2011 and -0.5 ± 0.1 μg/m^3^/year during 2011-2019. Trends before 2011 were driven (Fig. 3d) by marked increases in India (1.6 ± 0.2 μg/m^3^/year), China (1.5 ± 0.4 μg/m^3^/year), and other areas of South Asia (1.3 ± 0.3 μg/m^3^/year), and after 2011 by marked decreases in China (−2.4 ± 0.8 μg/m^3^/year), Eastern Europe (−0.6 ± 0.2 μg/m^3^/year), and Western Europe (−0.4 ± 0.1 μg/m^3^/year). Although these absolute trends are useful for comparing rates of change in pollution levels in each region, regional contributions to global net trends can be compared by also considering their relative population size.

**Fig. 4:**
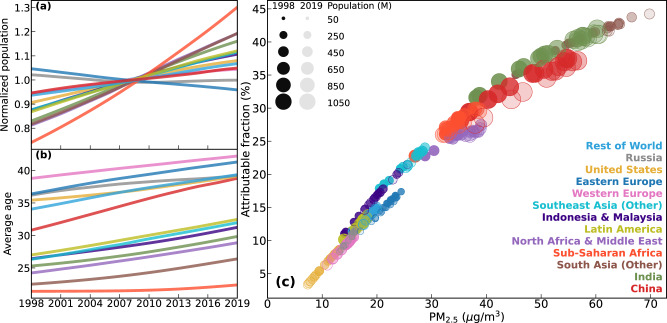
Changes in three factors altering PM_2.5_-attributable mortality over each region. **a** population (normalized to multi-year mean), (**b**) average age, (**c**) attributable fraction of baseline mortality to PM_2.5_ exposure. Colors represent different regions in each panel. In panel **c**, deaths from the six relevant diseases and ages <5 or ≥25are considered, size of each marker represents the regional population, and more recent years are indicated by more transparent colors.

We define and use normalized PW PM_2.5_ (“Methods” section, Eq. 8) to quantify the regional contributions to global PW PM_2.5_ in each year (Fig. 3a,b). In 1998, PW PM_2.5_ in China was 36.9 μg/m^3^, contributing to 27.8% (7.9 μg/m^3^) of global PW PM_2.5_ (28.3 μg/m^3^) after accounting for the 21.4% of global population from China. This contribution increases moderately to a peak value of 30.2% of global PW PM_2.5_ in 2006, weakly declines over 2006-2013, and then decreases substantially to 20.1% in 2019. Concurrently, the contribution from India increases rapidly from 22.4% in 1998 to 31.3% in 2019, driven by rapid increases in PW PM_2.5_ over 1998-2011 (Fig. 3d) and in population throughout 1998-2019 (Fig. 4a). The contribution of the entire South Asia region to global PW PM_2.5_ was 29.9% in 1998, greater than of China by just 2 percentage points, while increased to 40.9% in 2019, twice that of China. Another region with noteworthy increase in its contribution to global PM_2.5_ exposure is Sub-Saharan Africa, from 11.7% in 1998 to 13.1% in 2019. This region has the strongest growth rate in population (Fig. 4a), which led to reversal of the post-2011 decrease in PW PM_2.5_ (−2.5 μg/m^3^) to an increase (0.2 μg/m^3^, comparable to the decrease in India) in normalized PW PM_2.5_ (Fig. 3c). Conversely, regional contributions decreased by a factor of 2 or more over 1998-2019 for the US (2.1% to 0.9%), Western Europe (3.6% to 1.8%), Eastern Europe (2.7% to 1.2%), and Russia (1.5% to 0.7%). The latter two regions have population loss during 1998–2019 (Fig. 4a) which accelerated the reduction of this contribution.

Global mean PW PM_2.5_ increased by 10.5 μg/m^3^ from 1998 to 2011 (69% of this change coming from India and China), and decreased by 4.2 μg/m^3^ from 2011 to 2019 (3.8 μg/m^3^ from China) (Fig. 3c). Linearly fitting the regional time series of normalized PW PM_2.5_ (Fig. 3e) identifies India (0.31 ± 0.04 μg/m^3^/year) and China (0.24 ± 0.09 μg/m^3^/year) as the strongest contributors to the pre-2011 increase, followed by Sub-Saharan Africa (0.09 ± 0.04 μg/m^3^/year) and the rest of South Asia (0.08 ± 0.01 μg/m^3^/year). For the post-2011 decrease, China (−0.5 ± 0.1 μg/m^3^/year) alone is the dominant driver (Fig. 3c, e), followed by Western Europe (-0.03 ± 0.01 μg/m^3^/year), Southeast Asia (Other) (-0.02 ± 0.01 μg/m^3^/year) and Eastern Europe (−0.02 ± 0.01 μg/m^3^/year). Seasonal trends are further discussed in Supplementary Section S3 and Fig. S1, which reveal global trends and regional contributions that are overall consistent with the annual trends.

The analysis of yearly changes (Fig. 3c) and linearly fitted trends (Fig. 3e) quantitatively offer compelling evidence that the post-2011 decrease was largely driven by the decreasing PW PM_2.5_ exposure trend in China, and that since 2015 India has become the leading contributor to global ambient PM_2.5_ exposure. The different trajectories of population-weighted trends in China and India offer examples of how different air quality management strategies can induce pronounced air pollution changes within a short (<10 year) period. Nonetheless, the slowing growth of PM_2.5_ exposure in tropical regions should not be ignored. If the pre-2011 trends of PM_2.5_ exposure in each region had been sustained through 2019, the global PW PM_2.5_ would have increased by 6.5 μg/m^3^. In reality, global PW PM_2.5_ is 10.7 μg/m^3^ less relative to this crude “business as usual” case, with 5.5 μg/m^3^ contributed by China, 2.9 μg/m^3^ reduced in South Asia (including India), 1.1 μg/m^3^ alleviated in the Middle East and Africa, and 0.4 μg/m^3^ attributable to the two Southeast Asian regions. Relative to the scenario in which their PW PM_2.5_ concentrations remain stable at the 2011 level, the US and Europe contribute collectively to a 0.5 μg/m^3^ reduction of global PW PM_2.5_ in 2019.

Overall, using the decomposed regional contribution to global PM_2.5_ exposure, we find a determinant role of China as well as substantial contributions from tropical regions to the recent global reversal of trends in PM_2.5_ exposure.

### Stagnation of growth in PM_2.5_-attributable mortality

**Fig. 5:**
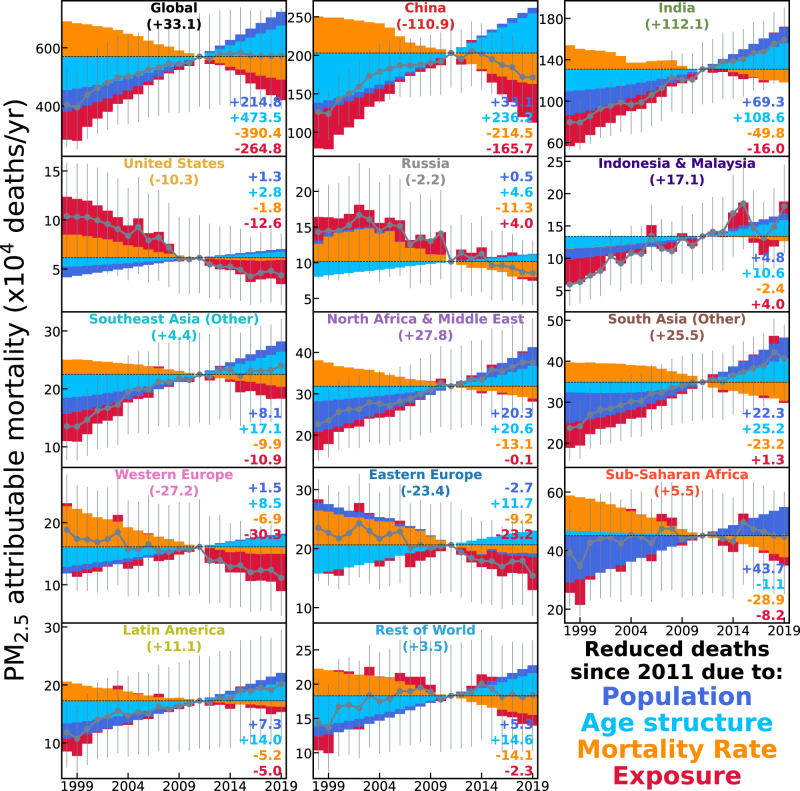
Trends in global and regional PM_2.5_ health burden, showing exposure and mortality mitigation counteracting population growth and aging. In each panel, gray points and lines indicate the time series of annual PM_2.5_-attributable mortalities (error bars represent the 95% confidence intervals), and colored bars represent changes (relative to 2011) attributed to exposure (dark red), baseline mortality rate (orange), population aging (light blue), and population growth (dark blue). Numbers inset at the right of each panel indicate the accumulated changes in mortality during 2012–2019 vs. 2011, colored by each of the four driving factors. Values following the region name at the top of each panel are the net changes during 2012–2019 summed from the four driving factors.

What are the associated health impacts of the changes in PW PM_2.5_ pollution presented above? We derive for each territory long-term changes of annual PM_2.5_-attributable mortality using the PW PM_2.5_ estimates, a concentration-response function (GEMM), and age- and disease-specific mortality data from the Global Burden of Disease (GBD) 2019 study (Methods). Figure 5 shows global and regional changes in annual PM_2.5_-attributable mortality during 1998–2019. Global annual attributable mortality increased steadily from 4.04 (95% CI: 2.59–5.33) million in 1998 to 5.70 (95% CI: 3.98–7.18) million in 2011. The pace slowed afterwards, peaking at 5.83 million (95% CI: 4.05–7.38) in 2015 and then decreasing slightly to 5.74 million (95% CI: 3.92–7.35) in 2019.

Relative to the pivot year of 2011, we use an order-based and year-by-year decomposition approach (Methods) to quantify the role of PM_2.5_ exposure and three other relevant demographic factors in driving the changes in annual PM_2.5_-attributable deaths. We extend previous decomposition studies that focused primarily on earlier time periods or differences between representative years^3,20^ to present contemporary global and regional time series of annual contributions to the changing mortality, especially those from changing PM_2.5_ exposure. A recent stagnation of global PM_2.5_-attributable deaths is identified and attributed to specific drivers by this continuous decomposition. Globally, annual PM_2.5_-attributable deaths increased by 0.97 million (0.78 million increase in India and China) from 1998 to 2011 and decreased by 0.70 million (0.50 million reduction in China) afterwards, due to changing PM_2.5_ exposure (dark red in Fig. 5, see also Supplementary Fig. S2a). Reductions in global PM_2.5_ exposure accumulated during 2012–2019 contribute to 2.65 million postponed PM_2.5_-attributable deaths, with 1.66 million (63%) from China. The larger contribution of other regions (e.g., the US and Europe) to reductions in mortality than to reductions in exposure reflect the increasing benefits of PW PM_2.5_ reduction at low PM_2.5_ concentrations and among older populations, as discussed in the next section. Western Europe (0.30 million), Eastern Europe (0.23 million), and the US (0.13 million) have the next highest exposure-driven reduced deaths after 2011 (25% in total), which are stronger than their contributions to the post-2011 reduction of global exposure (e.g., 15% in Fig. 3c).

In addition to exposure, three other factors also make sizable contributions to the changes in global PM_2.5_-attributable mortality (Supplementary Section S4). Globally, the 2.65 million reduced deaths by exposure reductions after 2011 are bolstered by those from reduced baseline mortality rates (3.90 million), to nearly offset the additional deaths due to population increases (2.15 million) and aging (4.74 million), yielding the net plateauing of growth observed in Fig. 5 (top left panel). Again, the achievement in China is noteworthy, with 1.10 million net reduced PM_2.5_-attributable deaths as accumulated since 2011 (1.20 million if benchmarked to 2013). The national reductions in China of annual PM_2.5_-attributable mortality and the determinant role of exposure reductions are consistent with findings of Geng et al.^26^ for 2012–2017. Here we further illustrate their driving role in the stagnation of growth in global PM_2.5_-attributable mortality. US and Europe contribute to an additional reduction of 0.61 million PM_2.5_-attributable deaths. Oppositely, strong net growth in annual mortality is found for India and the rest of South Asia (1.38 million excess deaths after 2011), followed by North Africa and the Middle East, Indonesia & Malaysia, and Latin America, due to insufficient reductions in PM_2.5_ exposure to counteract the increased mortality from population growth and aging.

Overall, our analysis reveals that globally, the slowing growth in PM_2.5_ exposure after 2011 critically offsets the effects on PM_2.5_-attributable mortality of a growing and aging global population, plateauing the previous growth in estimated global annual mortality caused by PM_2.5_ exposure.

### Increasing health benefits of PM_2.5_ reduction

**Fig. 6:**
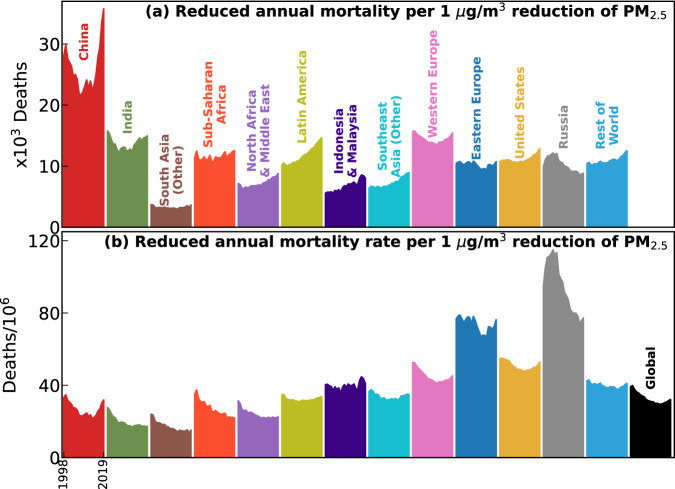
Global and regional changes in the marginal health benefits of PM_2.5_ mitigation. **a** Change of PM_2.5_-attributable deaths with 1 μg/m^3^ change in PM_2.5_ exposure in each region and year; (**b**) similar to **a** but normalized by relevant population (ages <5 or ≥25).

What level of health benefits does each region achieve by reducing the same amount of PW PM_2.5_, and how has this sensitivity changed as air quality and demography both evolve over time? Such insight from a global and long-term perspective is lacking while is particularly important to customize strategic mitigation policies at various levels of PM_2.5_ exposure. We derive a numeric representation of the changes in annual PM_2.5_-attributable deaths per 1 μg/m^3^ marginal reduction in PM_2.5_ exposure for each region and year, using a finite-difference approach (“Methods” section, Eqs. 12 and 13). China experienced the greatest marginal health benefits (i.e., reducing 21–36 thousand annual deaths) by mitigating 1 μg/m^3^ PM_2.5_ exposure among all regions (Fig. 6a), followed by Western Europe (13–16 thousand, with the highest average age, Fig. 4b) and India (12–16 thousand, with the second highest population). Large local population sizes contribute to these expected high benefits in these areas, as also suggested in previous studies^3,37^. In contrast, the per capita reduced deaths (Fig. 6b) are relatively higher (i.e., than the global mean level in black) over the US and Europe due to the more aged population (Fig. 4b), and over Russia due to the substantially higher baseline mortality rates (Supplementary Figs. S3a and S4).

**Fig. 7:**
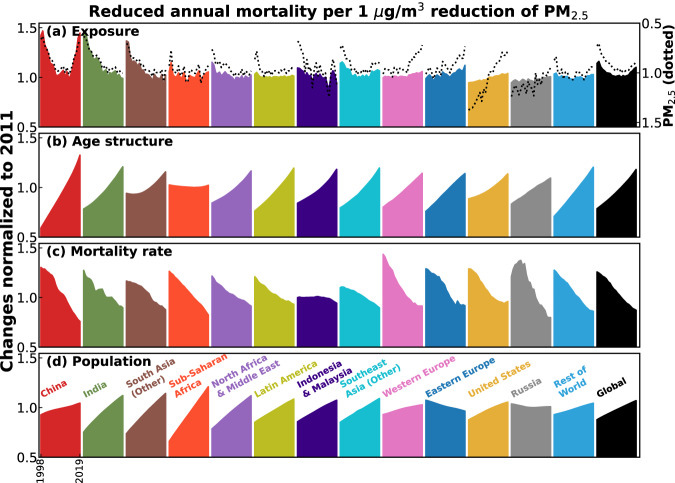
Decomposition of changes in marginal health benefits of PM_2.5_ mitigation. The sensitivity of PM_2.5_ attributable mortality to 1 μg/m^3^ difference in PM_2.5_ (normalized to 2011) over each region during 1998–2019 are decomposed to changes from (**a**) PM_2.5_ exposure, (**b**) population age structure, (**c**) baseline mortality rate, and (**d**) population growth. Dotted lines are the time series of population-weighted PM_2.5_ (also normalized to 2011) on a flipped *Y*-axis.

Globally, the per capita (for ages <5 or ≥25) health benefits per 1 μg/m^3^ marginal reduction (Fig. 6b, black) reduced from 39 to 30 annual deaths/million during 1998–2011, and then reversed to 32 annual deaths/million in 2019. All regions except Russia also experienced significantly (*p* < 0.05) increasing marginal health benefits since 2011 (Fig. 6a), because reductions in baseline mortality rates alone cannot offset the other three forces that largely enhanced the health benefits (Fig. 7 and Supplementary Section S5). Notably, the exposure-driven changes (Fig. 7a) were inversely related (*R*^2^ > 0.93) with PM_2.5_ exposure (dotted) in all of 13 regions, i.e., increasing PW PM_2.5_ leads to decreasing sensitivity of its attributable mortality and vice versa. These globally universal strong correlations were driven by the increasing sensitivity of the overall population attributable fraction (PAF) to PW PM_2.5_ changes at lower PW PM_2.5_ levels (Fig. 4c)^2,4,38^. Such a supra-linear exposure-response relationship has previously been discussed to elucidate greater health benefits from equally mitigated PW PM_2.5_ in less polluted regions of the world^10^, and in the cleaner future over the US^39^. This study provides a globally complete and long-term synthesis of this anti-correlation. The changes in PAF sensitivity are particularly strong at high PW PM_2.5_ before it stops increasing following lower PW PM_2.5_. Therefore, for most of the global population (e.g., 80% exposed to PM_2.5_ > 15 μg/m^3^ in 2019), mitigation of PW PM_2.5_ will continuously increase its health benefits at lower concentrations, and among older populations in the future.

### Implications

We were able to observe the worldwide reversal of exposure to PM_2.5_ air pollution, and quantify regional contributions to this long-term change, due to *a* > 15-year sustained effort to develop long-term high-resolution seamless observation-based global PM_2.5_ data. The estimates bridge the gap between ground monitoring of spatially discrete surface PM_2.5_ measurements and satellite observations of spatially continuous columnar aerosol optical depth (AOD), by leveraging state-of-science model representation of the PM_2.5_-AOD relationship and a powerful statistical tool to spatially propagate measurement information^28,40–42^. These satellite-derived PM_2.5_ estimates offer valuable data for vast global regions and population without regular monitoring of PM_2.5_ (over a billion persons in more than 100 territories worldwide^43^). Many poorly monitored areas (e.g., in Africa) exhibit dynamically varying changes in PM_2.5_ exposure (Fig. 2) and increasing contribution to global PM_2.5_ exposure (Fig. 3b), thus satellite-based estimates will need to be sustained in the future, apart from expansion of surface monitoring. Our analysis reports initial effects of recent environmental policies over South Asia, and cessation of growth in natural (e.g., dust and open fire) sources over the Middle East and Africa. Additional satellite-based and ground-level measurements in these regions are critical to monitoring future progress. A particular region of interest is Africa, where rapid population growth and industrialization are expected to pose stronger challenges to environmental regulations^44^ and potentially enhance its contribution to global PM_2.5_ pollution.

The normalized PW PM_2.5_ developed here defines the contribution to large-scale PM_2.5_ exposure from a specific location or region, enabling quantification of the importance of each region to the discovered global reversal. This metric is proportional to the product of (positively correlated) PM_2.5_ exposure and population, highlighting the significance and urgency of mitigating PM_2.5_ pollution over populous regions from another unique perspective. China was and India is now associated with >30% of global PM_2.5_ exposure, evidence of the critical roles played by population size and national air quality management strategies. The normalized PW PM_2.5_ metric is applicable to other underexploited studies, e.g., ranking the contribution to global PM_2.5_ pollution by populous cities, or comparison between rural vs. urban regions. Besides the global and regional insights in this paper, data of annual mean PM_2.5_ exposure, PM_2.5_-attributable mortality and marginal benefits, and their decompositions into drivers for all 204 territories are provided in an open-access data repository (see “Data Availability” Section) to support further analysis at smaller scales.

Two important findings in this comprehensive study support the need for additional future measures to further mitigate PM_2.5_ exposure worldwide: (1) the recent PW PM_2.5_ reductions globally and in many territories did not completely counteract the increase in global PM_2.5_-attributable mortality due to the growth and aging of population (Fig. 5), and (2) future health benefits are expected to increase more rapidly than in the past due to the globally universal stronger sensitivity of PAF at lower PW PM_2.5_ and the compounding effect of an ever-increasing and older population (Figs. 6 and 7). The latter perspective is timely especially considering the recent WHO update of the annual mean PM_2.5_ guideline^45^ (from 10 to 5 μg/m^3^). Emerging studies^38,46,47^ indicated even stronger and steeper concentration-response functions at low PW PM_2.5_ levels (<10 μg/m^3^), further strengthening the greater benefits at reduced PM_2.5_ exposure. However, future mitigation of PM_2.5_ air pollution at lower PM_2.5_ concentrations will be increasingly challenging, due to growing importance of aerosols arising from climate change (e.g., wildfire and dust)^32,36,48^, and as more readily mitigable anthropogenic sources are reduced^49,50^.

Based on available ground monitors, random uncertainties in the PM_2.5_ exposure estimates were found regionally variable while no systematic biases were identified (i.e., Fig. 1 and Supplementary Section S1), supporting the comparison of (normalized) PW PM_2.5_ and trends among regions. Supplementary Sections S6 and S7 (Figs. S5 and S6) further verified that our assessments and interpretations of trends in PM_2.5_ health impacts are overall insensitive to the choice of concentration response functions or decomposition approaches, although the estimated mortality numbers can significantly differ. This work also assumes equitoxicity of aerosol mass regardless of chemical mixture, the current approach employed by the GBD and WHO. This assumption may under- or over-estimate PM_2.5_-attributable mortality and its trends if such uniform toxicity were formally revised according to emerging evidence. Future advances in observational information and model capabilities to improve the accuracy of PM_2.5_ estimates, as well as updated PM_2.5_-health association from epidemiological studies, will reduce these uncertainties and support tracking the global evolution of PM_2.5_ air quality in response to air quality management and other emission changes, and the implications on human health.

## Methods

### Datasets

We use global satellite-derived PM_2.5_ estimates (V5.GL.03) available at monthly and annual temporal resolution over 1998-2019 and 0.01° × 0.01° (~1 × 1 km^2^) spatial resolution as described by van Donkelaar et al.^28^. Briefly, daily retrievals of columnar aerosol optical depth (AOD) were obtained from seven algorithms and four satellite instruments (Dark Target and Deep Blue for Terra- and Aqua-MODIS, MAIAC for combined Terra- and Aqua-MODIS, the Standard Aerosol algorithm for Terra-MISR, and Deep Blue for SeaStar-SeaWiFS). Each of these satellite AOD products and a chemical transport model (GEOS-Chem) simulation were compared with ground-based measurements of AOD from the global Aerosol Robotic Network (AERONET) to infer their biases and uncertainties. These daily AODs were then monthly averaged and combined using spatially resolved weights based on evaluation metrics, yielding best estimates of global monthly AOD on 0.01° × 0.01° grids through 1998-2019. The merged satellite observations comprise 91-96% of global population-weighted AOD for every month, with simulated GEOS-Chem AOD primarily used to fill the remaining grid cells (mostly snow covered) and produce the seamless AOD surface.

The best estimate AODs were then related to monthly PM_2.5_ using the GEOS-Chem simulation to account for the spatial and temporal variation of their relationship (*η*). *η* describes the state-of-science model representation of the processes affecting the AOD to PM_2.5_ relation including aerosol chemical composition, sampling time, hygroscopicity and vertical profile. These geophysical PM_2.5_ estimates explain about 80% of the variance in globally measured annual mean PM_2.5_ at surface monitoring sites collected by the World Health Organization (WHO)^42,51^.

To further improve the representation and fidelity of the PM_2.5_ estimates, ground-based observations of PM_2.5_ were collected and compiled from over 11,000 sites worldwide (e.g., Fig. 1a)^28^. Biases of geophysical PM_2.5_ estimates against these measured were interpreted as uncertainty sources of *η* from several relevant predictor variables, which were further parameterized and propagated to all grid cells using a geographically weighted regression^28,42^. These predicted biases were then applied to geophysical PM_2.5_ to provide the final hybrid estimates, which further increase the coefficient of determination (R^2^) of global annual PM_2.5_ measurements collected by the WHO (4409 sites) to 94%^28^. We use these hybrid estimates in this study.

We mainly use coefficient of determination (R^2^) and root mean square difference (RMSD, Eq. 1) to evaluate the estimates (${x}_{{est}}^{s}$) using collocated in situ (${x}_{{obs}}^{s}$) measurements (e.g., Extended Data Fig. 1b,c) across a total of *n* observational sites. RMSD is further normalized to the mean of monitor data to derive the normalized root mean square difference (NRMSD) in Eq. 2.

1 $${{{{{\rm{RMSD}}}}}}=\root {2} \of {\frac{{\sum }_{s=1}^{n}{\left({x}_{{est}}^{s}-{x}_{{obs}}^{s}\right)}^{2}}{n}},$$

2 $${{{{{\rm{NRMSD}}}}}}=\frac{{{{{{\rm{RMSD}}}}}}}{{\sum }_{s=1}^{n}{x}_{{obs}}^{s}/n}$$

We use age- and disease-specific number of deaths (i.e., baseline mortality) in 204 global countries and territories (collectively called “territories” in the paper) during 1998-2019, from the Global Burden of Disease (GBD) 2019 study. Six relevant diseases that contribute to PM_2.5_-attributable deaths are considered, including mortality from adult (25 years and older) with ischemic heart disease (IHD), Stroke, chronic obstructive pulmonary disease (COPD), lung cancer (LC), type-2 diabetes mellitus (DM), and childhood and adult (under 5 years and 25 years and older) acute lower respiratory infections (LRI). Deaths due to other non-communicable diseases are not considered in this study, which account for ~20% of total global PM_2.5_-attributable deaths^2^.

Age-specific population (*P*) counts for the 204 global territories are available for each year during 1998–2019 in the GBD 2019 dataset, which are combined with the age- and disease-specific deaths (*D*) to derive mortality rate (MR) and mean ages (Age_*m*_) for a region using Eqs. (3) and (4).

3 $${{{{{\rm{MR}}}}}}=\frac{{\sum }_{{age}}^{i}{\sum }_{{disease}}^{j}{\sum }_{{territory}}^{k}{D}_{i,\, j,\, k}}{{\sum }_{{age}}^{i}{\sum }_{{territory}}^{k}{P}_{i,\, k}},$$

4 $$\,{{{{{{\rm{Age}}}}}}}_{m}=\frac{{\sum }_{{age}}^{i}{\sum }_{{territory}}^{k}{{{{{{{\rm{Age}}}}}}}_{i,\, k}\times P}_{i,\, k}}{{\sum }_{{age}}^{i}{\sum }_{{territory}}^{k}{P}_{i,\, k}},$$

The GBD population data for each territory contains 96 age bins, with population number for ages with <1, 1, 2,…, 94, and 95+ years. We assign an age of 0 to the <1 year group, and of 95 to the 95+ years group.

Spatially resolved population estimates at 30-s resolution (~1 km) are from the Gridded Population of the World (GPW v4) database. GPW is available every five years for 2000–2020. For each year during 1998–2019, we extract the GPW population in the closest year and regridded the data into regular 0.01° × 0.01° grids. These regridded population distributions are further scaled for each of the 204 territories to match their total populations for that year in the GBD 2019 population data.

### Population-weighted PM_2.5_ and its normalization

The high-resolution GPW population estimates (*P*) are used with the PM_2.5_ estimates (both 0.01° × 0.01°) to derive PW PM_2.5_ exposure (*E*) for each territory (region) and year based on the gridded PM_2.5_ (PM_*l*_) from each grid cell location (*l*).

5 $$\,E=\frac{{\sum }_{{location}}^{l}{{{{{{{\rm{PM}}}}}}}_{l}\times P}_{l}}{{\sum }_{{location}}^{l}{P}_{l}}$$

We evaluate the PW PM_2.5_ estimates using a bootstrapping approach (e.g., Fig. 1d). For each region with a total of *N* ground sites, we randomly select *n* sites to calculate PW PM_2.5_ from both the ground-based observations and the collocated estimates (*n* varies from 10 to 110). We repeat the random sampling *m* times, where $m=N/n\times 200$, and thus get *m* collocated pairs of observed and estimated PW PM_2.5_, which are then used to calculate the NRMSD in Fig. 1d.

It can be inferred from Eq. 5 that the global PW PM_2.5_ can be calculated as in Eq. 6.

6 $${E}_{{global}}=\frac{{\sum }_{{region}}^{k}{{E}_{k}\times P}_{k}}{{\sum }_{{region}}^{k}{P}_{k}}$$

Therefore, contributions from each region to the global PW PM_2.5_ can be expressed as the sum of normalized PW PM_2.5_ (NE).

7 $${E}_{{global}}=\mathop{\sum }\limits_{{region}}^{k}{{{{{{\rm{NE}}}}}}}_{k}$$

8 $${{{{{{\rm{NE}}}}}}}_{k}=\frac{{{E}_{k}\times P}_{k}}{{\sum }_{{region}}^{k}{P}_{k}},$$

Increases in normalized PW PM_2.5_ of a region thus can be from increases in either its PM_2.5_ exposure or its population share of the world.

### Trend estimates

We estimate linear trends in the (normalized) PW PM_2.5_ time series for each territory (Fig. 2) and region (Fig. 3d, e), based on a linear least square fitting approach. We report the linear slope (concentration trend in μg/m^3^/year), its 95% confidence interval (CI), as well as the *p*-value (two-tailed Student’s *t* test). In our analysis, slopes with *p*-value of <0.05 are associated with their 95% CIs not enveloping the zero point and vice versa, therefore the *p*-values and 95% CIs have equivalent indications on the significance of derived trends. These multi-year slopes are less sensitive to abnormal years, relative to differences between two benchmark years. We also confirmed that the derived trends in PW PM_2.5_ of the 204 territories are highly consistent (*R*^2^ > 0.9) with those from non-parametric (Mann-Kendall^52,53^) trend estimation approach.

### PM_2.5_-attributable mortality and its sensitivity to PM_2.5_ mitigation

For each territory, we calculate its annual PM_2.5_-attributable deaths (*D*_*PM*_) based on Eqs. (9) and (10) and the changing baseline mortality data and PW PM_2.5_ in each year.

9 $${D}_{{PM}}=\mathop{\sum }\limits_{{age}}^{i}\mathop{\sum }\limits_{{disease}}^{j}{D}_{i,\, j}\times {{{{{{\rm{PAF}}}}}}}_{i,\, j}(E)$$

10 $$\,{{{{{{\rm{PAF}}}}}}}_{i,\, j}(E)=(1-\frac{1}{{{{{{{\rm{RR}}}}}}}_{i,\, j}(E)})$$

In this study, the relative risk (RR) as the concentration response function (CRF) of PM_2.5_ exposure (*E*) is adopted for each age group and disease from the Global Exposure Mortality Model (GEMM)^2^, to derive the population attributable fraction (PAF) as in Eq. 10. GEMM provides age-specific CRFs for IHD and stroke (i.e., 25 and over at 5-year interval steps), and age-independent CRFs for COPD, LC, DM (≥25 years) and LRI (all ages). All the GEMM CRFs are derived directly from studies of health impacts of outdoor exposure to PM_2.5_, and are applied after dividing by the RR at Theoretical Minimum Risk Exposure Level, consistent with McDuffie et al.^13^.

We obtain the GEMM CRFs and their 95% CIs from a public accessible repository^54^. We follow the conventional approach^1,2,13^ to calculate 95% CIs of PM_2.5_-attributable deaths, by applying the upper and lower bounds in the 95% CIs of the CRFs to Eq. 10. We verified that these derived uncertainty bounds encompass the uncertainty ranges due to the 95% CIs of the baseline mortality, or of the PW PM_2.5_ (assumed 40% for Africa and the Middle East and 20% for other regions, roughly 2 times the NRMSD in Fig. 1d). As the 95% CIs in the baseline mortality, CRFs and PW PM_2.5_ are all estimated from a bootstrapping approach, quadratically propagating the uncertainties from these variables likely leads to an overestimate in the breadth of the 95% CI for the total attributable disease burden if these error sources are correlated.

We examined the sensitivity of our results to the choice of mortality relative risk model by re-estimating attributable mortality with the Meta Regression-Bayesian, Regularized, Trimmed (MR-BRT) CRFs used by the GBD Collaboration^1,20,55^. We found that the results from the GEMM-based analyses (Fig. 5), e.g., the cessation of growth of the health burdens in recent years, the dominant reductions from China, and competition of the four drivers were largely consistent when the MR-BRT function was used (see Supplementary Section S6 and Fig. S5) despite differences in model assumptions and applicability.

It is instructive to derive the overall PAF (PAF_*a*_) for a territory or region that accounts for all relevant ages and diseases (e.g., Fig. 4c):

11 $${{{{{{\rm{PAF}}}}}}}_{a}=\frac{{\sum }_{{age}}^{i}{\sum }_{{disease}}^{j}{D}_{i,\, j}\times {{{{{{\rm{PAF}}}}}}}_{i,\, j}}{{\sum }_{{age}}^{i}{\sum }_{{disease}}^{j}{D}_{i,\, j}},$$

We further derive the sensitivity of *D*_*PM*_ to changes in PW PM_2.5_ (e.g., Fig. 6) as Eq. 12.

12 $$\,\frac{\Delta {D}_{{PM}}}{\Delta E}=\mathop{\sum }\limits_{{age}}^{i}\mathop{\sum }\limits_{{disease}}^{j}{D}_{i,j}\times \frac{\Delta {{{{{{\rm{PAF}}}}}}}_{i,\, j}}{\Delta E}$$

We use a finite-difference approach (Eq. 13) to calculate the sensitivity of PAF to PM_2.5_ ($\frac{\Delta {{{{{{\rm{PAF}}}}}}}_{i,j}}{\Delta E}$), in which the PW PM_2.5_ is varied by 5% ($\Delta E=5\%E$) in positive and negative directions to calculate the changes in PAF:

13 $$\,\frac{\Delta {{{{{{\rm{PAF}}}}}}}_{i,\, j}}{\Delta E}=\frac{{{{{{{\rm{PAF}}}}}}}_{i,\, j}(E+\Delta E)-{{{{{{\rm{PAF}}}}}}}_{i,\, j}(E-\Delta E)}{2\Delta E}$$

The finite-difference of exposure ($2\Delta E$) is constrained to be at least 1 μg/m^3^, and at most 5 μg/m^3^. Our sensitivity tests (e.g., $\Delta E=10\%E$ or changing the stratified values) verified that the derived $\frac{\Delta {D}_{{PM}}}{\Delta E}$ are not sensitive to the choice of finite-differences.

### Attribution of changes in PM_2.5_-attributable mortality to drivers

For each territory *k*, age *i* and disease *j*, the corresponding PM_2.5_ attributable deaths *D*_*PM*_ (Eq. 9) can be expanded to the product of four parameters:

14 $$\,{D}_{{PM}}(i,\, j,\, k)={D}_{i,\, j,\, k} * {{{{{{\rm{PAF}}}}}}}_{i,\, j}={P}_{k}\times {{{{{{\rm{AF}}}}}}}_{i,\, k}\times {{{{{{\rm{MR}}}}}}}_{i,\, j,\, k}\times {{{{{{\rm{PAF}}}}}}}_{i,\, j}({E}_{k})$$

where *P*_*k*_ is the total population, AF_*i,k*_ is its fraction of population within age *i* (representing age structure), MR_*i,j,k*_ is mortality rate of disease *j* within age *i* for territory *k*, and PAF is a monotonic function of PM_2.5_ exposure (*E*). For two neighboring years (*y* and *y* + *1*), the changes in annual deaths can be attributed to changes in these four factors.

Following Geng et al.^26^, the differences in *D*_*PM*_ between year and year+1 can be expanded as fractional differences by gradually varying the four factors from year to year+1:

15 $${D}_{{{{\rm{PM}}}}}^{{{{\rm{yr}}}}+1}-{D}_{{{{\rm{PM}}}}}^{{{{\rm{yr}}}}}=	{{D}_{{{{\rm{PM}}}}}\left({P}^{{{{\rm{yr}}}}+1},{{{{\rm{AF}}}}}^{{{{\rm{yr}}}}+1},{{{{\rm{MR}}}}}^{{{{\rm{yr}}}}+1},{{{{\rm{PAF}}}}}^{{{{\rm{yr}}}}+1}\right)-D}_{{{{\rm{PM}}}}}\left({{{{\rm{P}}}}}^{{{{\rm{yr}}}}},{{{{\rm{AF}}}}}^{{{{\rm{yr}}}}},{{{{\rm{MR}}}}}^{{{{\rm{yr}}}}},{{{{\rm{PAF}}}}}^{{{{\rm{yr}}}}}\right)\\=	{D}_{{{{\rm{PM}}}}}\left({P}^{{{{\rm{yr}}}}+1},{{{{\rm{AF}}}}}^{{{{\rm{yr}}}}+1},{{{{\rm{MR}}}}}^{{{{\rm{yr}}}}+1},{{{{\rm{PAF}}}}}^{{{{\rm{yr}}}}+1}\right)-{D}_{{{{\rm{PM}}}}}\left({P}^{{{{\rm{yr}}}}},{{{{\rm{AF}}}}}^{{{{\rm{yr}}}}+1},{{{{\rm{MR}}}}}^{{{{\rm{yr}}}}+1},{{{{\rm{PAF}}}}}^{{{{\rm{yr}}}}+1}\right)\\ 	+{D}_{{{{\rm{PM}}}}}\left({P}^{{{{\rm{yr}}}}},{{{{\rm{AF}}}}}^{{{{\rm{yr}}}}+1},{{{{\rm{MR}}}}}^{{{{\rm{yr}}}}+1},{{{{\rm{PAF}}}}}^{{{{\rm{yr}}}}+1}\right)-{D}_{{{{\rm{PM}}}}}\left({P}^{{{{\rm{yr}}}}},{{{{\rm{AF}}}}}^{{{{\rm{yr}}}}},{{{{\rm{MR}}}}}^{{{{\rm{yr}}}}+1},{{{{\rm{PAF}}}}}^{{{{\rm{yr}}}}+1}\right) \\ 	+{D}_{{{{\rm{PM}}}}}\left({P}^{{{{\rm{yr}}}}},{{{{\rm{AF}}}}}^{{{{\rm{yr}}}}},{{{{\rm{MR}}}}}^{{{{\rm{yr}}}}+1},{{{{\rm{PAF}}}}}^{{{{\rm{yr}}}}+1}\right)-{D}_{{{{\rm{PM}}}}}\left({P}^{{{{\rm{yr}}}}},{{{{\rm{AF}}}}}^{{{{\rm{yr}}}}},{{{{\rm{MR}}}}}^{{{{\rm{yr}}}}},{{{{\rm{PAF}}}}}^{{{{\rm{yr}}}}+1}\right) \\ 	+{D}_{{{{\rm{PM}}}}}\left({P}^{{{{\rm{yr}}}}},{{{{\rm{AF}}}}}^{{{{\rm{yr}}}}},{{{{\rm{MR}}}}}^{{{{\rm{yr}}}}},{{{{\rm{PAF}}}}}^{{{{\rm{yr}}}}+1}\right)-{D}_{{{{\rm{PM}}}}}\left({P}^{{{{\rm{yr}}}}},{{{{\rm{AF}}}}}^{{{{\rm{yr}}}}},{{{{\rm{MR}}}}}^{{{{\rm{yr}}}}},{{{{\rm{PAF}}}}}^{{{{\rm{yr}}}}}\right)\\=	 \delta {D}_{{PM}}^{1}(P)+\delta {D}_{{PM}}^{1}({{{\rm{AF}}}})+\delta {D}_{{PM}}^{1}({{{\rm{MR}}}})+\delta {D}_{{PM}}^{1}({{{\rm{PAF}}}})$$

The superscript 1 indicate that this is 1 of 24 (=4 × 3 × 2 × 1) possible pathways (as altering the order of changing the four factors) that the fractional differences due to each factor can be derived. The final attributed differences due to population change is the ensemble mean of the 24 scenarios:

16 $$\,\delta {D}_{{PM}}(P)=\frac{{\sum }_{t=1}^{24}{\delta D}_{{PM}}^{t}(P)}{24}$$

The attribution to the other three factors is similarly performed.

We apply this method to every pair of neighboring years to minimize the effects of nonlinearity. These attributed differences are then summed across diseases, ages, and territories, and finally all accumulated referenced to 2011, to derive the regional attributions (e.g., Fig. 5). We verify that the decomposition approach is robust as exhibiting a high degree of consistency with an alternative method (e.g., Supplementary Section S7 and Fig. S6).

The attribution of changes in $\frac{\Delta {D}_{{PM}}}{\Delta E}$ is similarly performed (e.g., Fig. 7), with the PAF in Eq. 15 replaced with $\frac{\Delta {{{{{\rm{PAF}}}}}}}{\Delta E}$ which also represents the changes due to PM_2.5_ exposure.

### Supplementary information

Supplementary Information for

Peer Review File

## Data Availability

Source data are provided with this paper. All input data necessary to replicate the analysis results are deposited to a Zenodo repository: 10.5281/zenodo.7618789, with detailed supporting documentation.

## Code availability

All analyses and visualizations in this manuscript are facilitated by codes and data deposited to 10.5281/zenodo.7618789, with detailed supporting documentation. All the maps in this manuscript are generated using open-access python packages cartopy (https://scitools.org.uk/cartopy/) and matplotlib (https://matplotlib.org).
