# Supplementary material for: Reversal of trends in global fine particulate matter air pollution
Source: Nat Commun. 2023 Sep 2;14:5349. doi: 10.1038/s41467-023-41086-z (PMC10475088; doi:10.1038/s41467-023-41086-z)
Supplement: Supplementary file 1 — Supplementary Information for [file 41467_2023_41086_MOESM1_ESM.pdf]

Supplementary Information for "Reversal of trends in global fine particulate matter air pollution"

Chi Li[1*], Aaron van Donkelaar[1], Melanie S. Hammer[1], Erin E. McDuffie[1,11], Richard T. Burnett[2,3], Joseph V. Spadaro[4,5], Deepangsu Chatterjee[1], Aaron J. Cohen[6,2], Joshua S. Apte[7,8], Veronica A. Southerland[9], Susan C. Anenberg[9], Michael Brauer[10,2], Randall V. Martin[1]

1. Department of Energy, Environmental & Chemical Engineering, Washington University in St. Louis, St. Louis, MO, USA

2. Institute for Health Metrics and Evaluation, University of Washington, Seattle, WA, USA

3. Population Studies Division, Health Canada, Ottawa, ON, Canada

4. Spadaro Environmental Research Consultants (SERC), Philadelphia, PA, USA

5. European Centre for Environment and Health, World Health Organization (Consultant), Bonn, North Rhine-Westphalia, Germany

6. Health Effects Institute, Boston, MA, USA

7. Department of Civil and Environmental Engineering, University of California, Berkeley, CA, USA

8. School of Public Health, University of California, Berkeley, Berkeley, CA, USA

9. Milken Institute School of Public Health, George Washington University, Washington DC, USA

10. School of Population and Public Health, University of British Columbia, Vancouver, BC, Canada

11. Now at: Office of Atmospheric Protection, Climate Change Division, U.S. Environmental Protection Agency, Washington, D.C., USA

*Correspondence to chili@wustl.edu

S1. Evaluation of satellite-derived $PM_{2.5}$

Annual mean $PM_{2.5}$ concentrations from the satellite-derived estimates span across two orders of magnitude and are consistent (R^2=0.90 in 2017) with in situ observations worldwide (Fig. 1b). The consistency is stable across 2015-2019 (Fig. 1c) when available observational sites significantly expanded globally (Fig. 1a). Notably, these agreements also exhibit distinct regional variation (R^2 of 0.4-0.9 and normalized root mean square difference NRMSD of 15-50%, Fig. 1c) due to regional variability in the availability and reliability of information to constrain and evaluate the estimates[1]. The agreement further improves when evaluated over a wider area using number of sites as a proxy of averaged domain breath (Fig. 1d), providing a measure of confidence in the area-aggregated analysis examined here. All regions exhibit NRMSD of 5-10% when >100 sites are averaged except for the Middle East and Africa (NRMSD ~20%). These NRMSDs diminish more slowly as sample size exceeds 50, and therefore constrain the upper bounds of the uncertainties in the regional population-weighted (PW) $PM_{2.5}$ estimates.

S2. Reversal of $PM_{2.5}$ exposure trends worldwide

Regional changes in (PW) $PM_{2.5}$ over North America, Europe, China and South Asia have been individually investigated[1–8], with anthropogenic emission changes driven by socio-economic developments and environmental policy attributed as the main driver. Since 2011, the reduction of growth rates of PW $PM_{2.5}$ across South Asia is especially noteworthy. A recent observation-based study[7] reported significant reductions of mean $PM_{2.5}$ and $PM_{2.5}$ exceedance days over five megacities of India during 2014-2019, and attributed these unprecedented trends to clean air measures mainly implemented in urban areas of India[9]. The satellite-derived estimates further identify a reduction in PW $PM_{2.5}$ growth rates across the broader South Asian region. Several territories in East Asia (e.g., Japan and North Korea) and Southeast Asia (e.g.,

Vietnam, Thailand and Philippines) also exhibit pronounced reversal of PW $PM_{2.5}$ trends, which relates to local pollution abatement actions[10–13] as well as reductions or slowed growth of transboundary pollutants from China and India. Zhang et al.[14] estimated that emissions from China are the strongest non-local source of $PM_{2.5}$-attributable deaths in these five territories, e.g., 40.5% in the rest of East Asia.

Across other regions of the Global South (the Middle East, Africa and South America), mineral dust and biomass burning smoke are more dominant in $PM_{2.5}$ exposure[15,16] and may influence its trends[17]. Examples in our estimates include the steady increase of dust emission in the Middle East before 2011[3,18] (Fig. 2a) that weakened afterwards, and the recent decline of burned area and fire-driven $PM_{2.5}$ in Central Africa[19,20] (Fig. 2b). Open burning is also the dominant source of $PM_{2.5}$ concentrations across the Amazon, which were reduced over 2001-2012 by governmental actions to contain deforestation[21]. These actions were also counteracted by frequent occurrences of droughts[21,22], causing overall insignificant trends in PW $PM_{2.5}$ (Fig. 2). Stronger measures may be needed to compensate for climate-induced variability and more effectively mitigate $PM_{2.5}$ for these regions in the future.

S3. Regional contributions to global changes in $PM_{2.5}$ exposure in four seasons

As most of the substantial changes in PW $PM_{2.5}$ occur in the populous Northern Hemisphere (NH), global PW $PM_{2.5}$ magnitude and most regional changes are strongest in the season of NH winter (DJF, Fig. S1), when multiple meteorological (e.g., inversions and shallow boundary layer[23,24]), chemical (e.g., heterogeneous haze formation[25,26]) and anthropogenic (e.g., residential heating[15,27]) factors collectively facilitate a seasonal tendency for elevated $PM_{2.5}$. Nevertheless, the reversal of global PW $PM_{2.5}$ trends and the driving roles of China and India persist across all four seasons (Fig. S1).

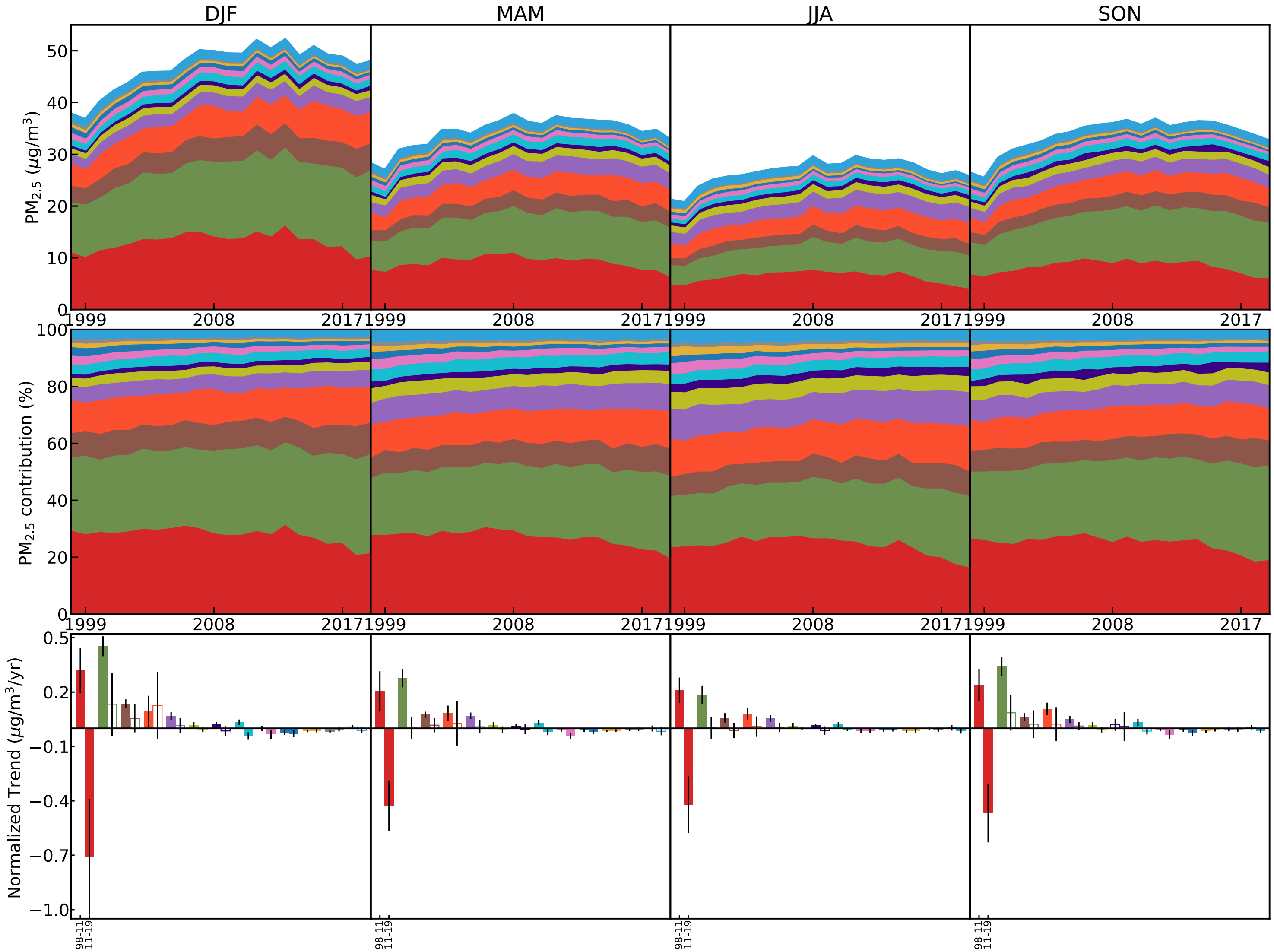

Figure S1. Absolute (upper row) and relative (middle row) contribution from 13 regions (color-coding consistent with the main text) to global population-weighted $PM_{2.5}$ changes in northern hemispheric winter (DJF), spring (MAM), summer (JJA) and fall (SON). The lower row shows trends (error bars indicating 95% confidence intervals) derived from these normalized time series for each region over 1998-2011 and 2011-2019.

S4. Changes in $PM_{2.5}$-attributable mortality due to non-$PM_{2.5}$ factors

After the decomposition (Methods), changes in baseline mortality rate (i.e., orange in Fig. 5) following improvements in medical care and well-being induce continuous decreases while population growth and aging oppositely contribute to increases (Figs. 5 and S2b-d). Meanwhile, certain factors are more pronounced in different regions due to variations in local exposure level and demography. For example, population growth (i.e., dark blue in Fig. 5) occurred almost everywhere (except for Russia and Eastern Europe), contributing to increases in the $PM_{2.5}$-attributable deaths and ranking as the dominant driver in Sub-Saharan Africa (Figs. 4a and 5). Population aging (i.e., light blue in Fig. 5) is the strongest driver globally and in China, where mean population age increases substantially to the level similar to that in the US in 2019 (Fig. 4b). In developed regions of the US and Europe, $PM_{2.5}$ exposure becomes the dominant driver of net changes in annual burden especially since 2011 (Fig 5), as changes in the other three factors are slowed/saturated following socioeconomic developments (e.g., Figs. 4, S3, S4). These regionally varying contributions to changing $PM_{2.5}$-attributable mortality are overall consistent with previous global-scale studies[6,28]. Our unique insights from the annual time series extend beyond the inflection in exposure to also include the evolution of contributions from the other three demographic factors relative to that of $PM_{2.5}$ exposure (Fig. 5), which has stronger interannual variability especially over regions significantly affected by mineral dust and biomass burning smoke (e.g., the Middle East, Africa, Indonesia & Malaysia, Russia and Latin America).

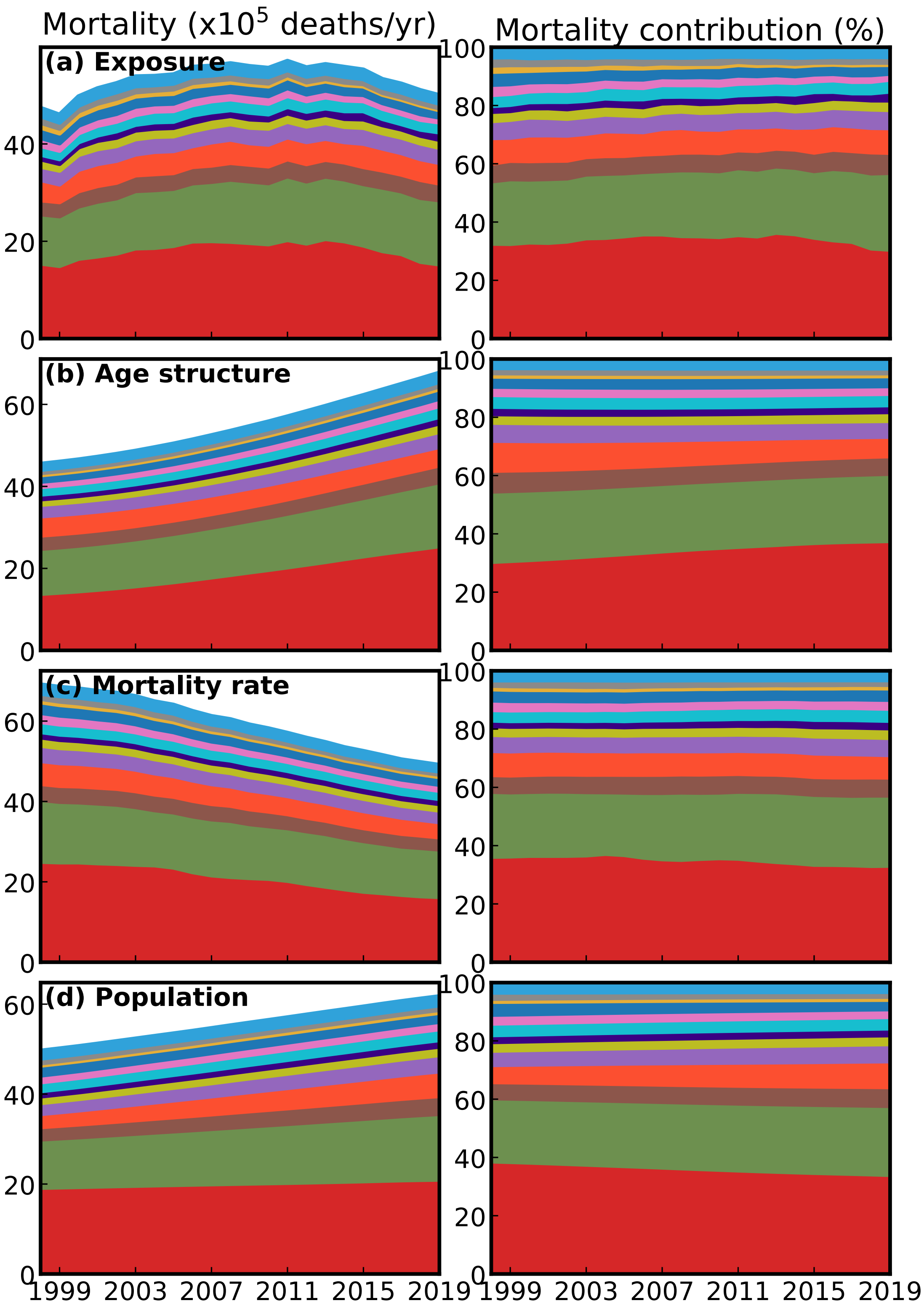

Figure S2. Absolute (left) and relative (right) regional contributions to changes in $PM_{2.5}$-attributable mortality as caused by changes in (separated by rows) $PM_{2.5}$ exposure (a), population age structure (b), baseline mortality rate (c), and population (d). Changes (relative to 2011) due to each factor are combined with the $PM_{2.5}$-attributable mortality in 2011 to produce the mortality time series for each region (color-coding consistent with the main text).

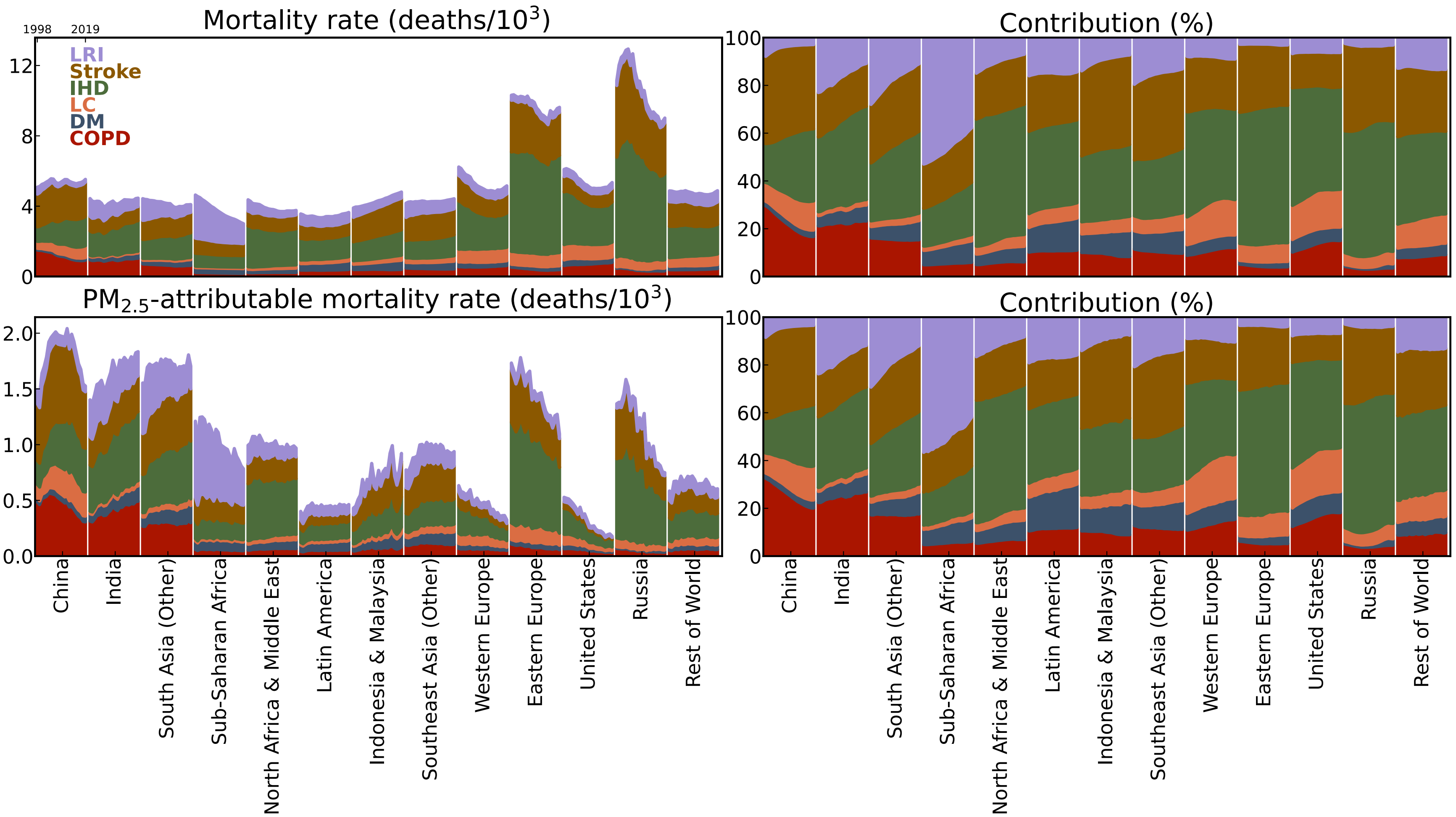

Figure S3. Changes in baseline (upper) and $PM_{2.5}$-attributable (lower) mortality rates in each region and the absolute (left) and relative (right) contributions of the six diseases (color-coded). Deaths from ages <5 or ≥25 are considered.

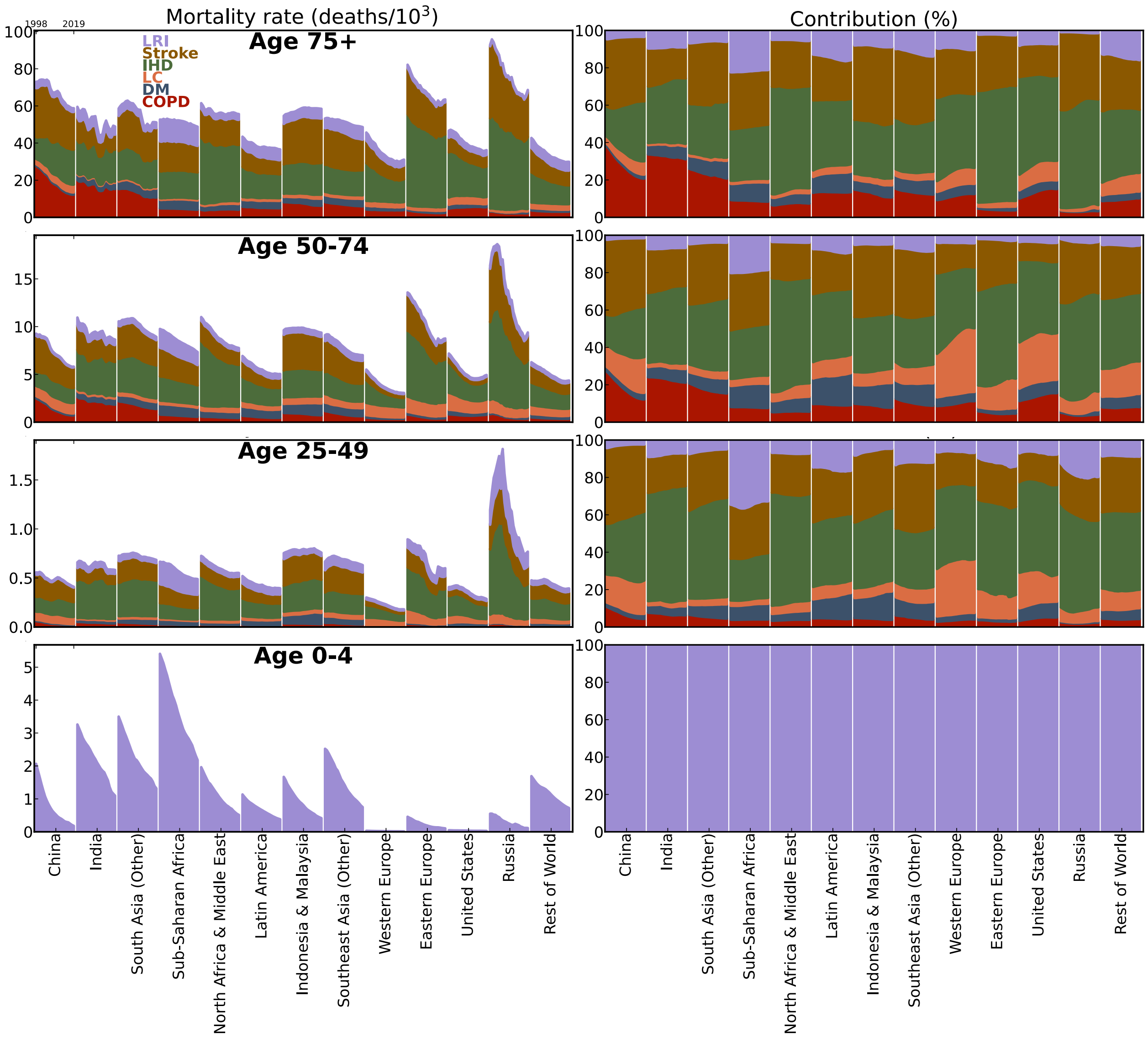

Figure S4. Changes in baseline mortality rates in each region and the absolute (left) and relative (right) contributions of the six diseases (color-coded) in separate age groups.

S5. Changes in marginal health benefits of $PM_{2.5}$ mitigation due to non-$PM_{2.5}$ factors

Variation exists in the degree of changes in calculated marginal health benefits due to non-$PM_{2.5}$ factors (Fig. 7b-d). Population aging has enhanced the health benefits of PW $PM_{2.5}$ reductions in most regions during the past two decades (Fig. 7b), and is also the strongest driver of the post-2011 net increases globally (Fig. 7, black). Pronounced increases of this sensitivity to average age (Fig. 7b) occurred in China with the strongest increase in average age, and in Europe

with the highest average age (Fig. 4b). In contrast, sensitivity to population aging is substantially weaker in South Asia, the Middle East and Africa where the average age is >10 yrs lower than in Europe (Fig. 4b). This contrast reflects the orders-of-magnitude stronger baseline mortality rates in the elderly population (Fig. S4). Nonetheless, population aging is projected across the world (e.g., Figs. 4b and 7b)[29,30], and is expected to strengthen the benefits and significance of PW $PM_{2.5}$ mitigation even over these "younger" regions in the future.

Changes in baseline mortality rates (Fig. 7c) and population (Fig. 7d) in general induced opposite effects on the health benefits, with exceptions in Eastern Europe and Russia where populations are declining (Fig. 4a). In developed regions of the US and Europe, the reductions driven by decreasing baseline mortality rates substantially slowed in recent years, implying the need for further PW $PM_{2.5}$ reductions to reduce its attributable mortality in these regions (e.g., Figs. S3, S4). On the other hand, in Sub-Saharan Africa the strongest regional population growth induces the most substantial increase in the health benefits of PW $PM_{2.5}$ reduction.

S6. Sensitivity calculation using the MR-BRT CRFs

The Meta Regression-Bayesian, Regularized, Trimmed (MR-BRT) CRFs were used in the GBD 2019 study to estimate $PM_{2.5}$ health burden from both ambient and household $PM_{2.5}$ pollutions. Unlike the GEMM that is purely based on studies of outdoor $PM_{2.5}$ health impacts, MR-BRT is synthesized from cohort and case-control studies of outdoor $PM_{2.5}$, household pollution due to the use of solid fuel, and secondhand smoke[31]. Directly combining ambient $PM_{2.5}$ exposure with the MR-BRT CRFs yields the health burden due to both ambient $PM_{2.5}$ pollution and co-exposure to household $PM_{2.5}$. A way to isolate outdoor exposure in the MR-BRT derived $PM_{2.5}$-attributable mortality is to apply co-exposure adjustment factors that are territory- and year-dependent[15].

Fig. S5 (grey circles with error bars) presents the time series and 95% CIs of annual $PM_{2.5}$-attributable deaths using our exposure estimates and the MR-BRT CRFs which are decomposed to the changes due to each driver similar to Fig. 5. The MR-BRT CRFs suggests similar level of health risk of $PM_{2.5}$ at relatively low $PM_{2.5}$ exposure (e.g. < 25 $\mu g/m^3$) while substantially lower health risk at higher PW $PM_{2.5}$ levels[15,32]. As a result, the derived deaths agree in magnitudes with those from the GEMM (Fig. 5) over the US, Europe and Russia, while are lower by ~25% over China and South Asia. We note that the relative risk (*RR*) values in MR-BRT at PW $PM_{2.5}$>35 $\mu g/m^3$ are constrained by studies of household or secondhand smoke pollution[31], while GEMM filled this gap of data by incorporating a cohort study of outdoor $PM_{2.5}$ impacts in China[32].

While the MR-BRT derived $PM_{2.5}$-attributable deaths support the decomposition and analysis in Fig. S5, they still contain the above-mentioned co-exposure. We thus also include in Fig. S5 the annual deaths and 95% CIs after applying the co-exposure adjustment factors (grey dashed lines and shadings), and from the GBD 2019 study (gold dotted lines and shadings). The co-exposure factors are derived to scale the MR-BRT deaths calculated with the ambient $PM_{2.5}$ exposure in GBD 2019 to match the GBD reported deaths for each territory and year, so the small and insignificant differences between the dashed grey (from our study) and dotted gold (from GBD 2019) are due solely to differences in the exposure data used. The deaths due to ambient exposure only (dashed grey) overlap with those of total deaths (solid grey) over the US and Western Europe due to the negligible amount of solid fuels used in these regions, while are significantly lower in Asia and Africa. The gap is substantially narrowed over China in recent years, reflecting the progress of regulating household use of solid fuels[33].

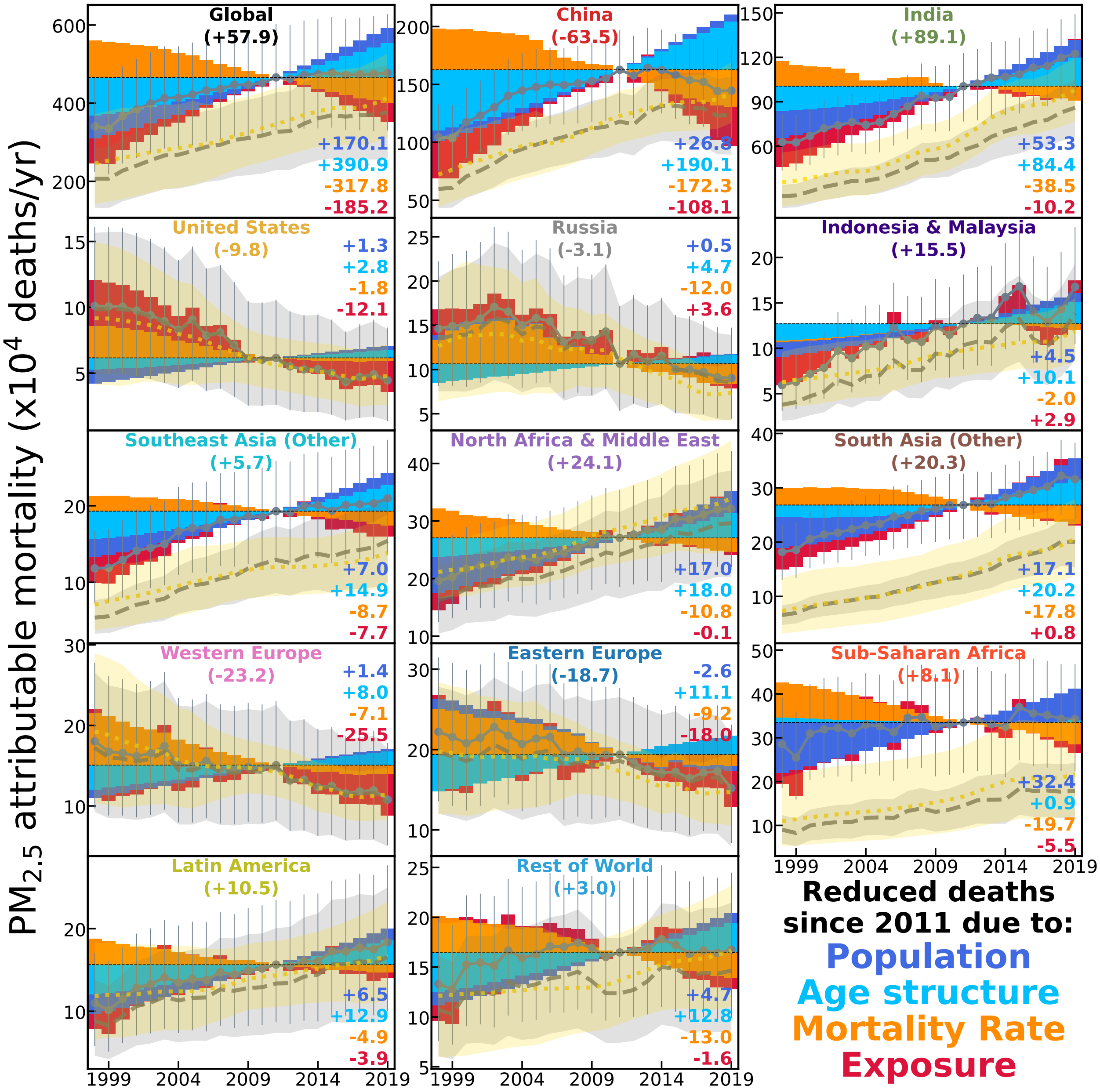

Figure S5. Similar to Figure 5 but using the MR-BRT concentration-response functions. Grey dashed lines (with the light grey envelope indicating the 95% confidence interval) represent the annual time series of $PM_{2.5}$-attributable mortality after applying the indoor and outdoor co-exposure factors. Gold dotted lines (with the light gold envelope indicating the 95% confidence interval) represent the annual time series of $PM_{2.5}$-attributable mortality from the GBD-2019 study.

Despite the above issues that inhibit the use of the MR-BRT model for our analysis, the overall conclusions in the GEMM results (Fig. 5), e.g., the cessation of growth in recent years and the dominant reductions from China, are consistently present in both the total deaths (solid

grey) and the deaths due to outdoor exposure only (dashed grey) from the MR-BRT model. The inflection year (e.g., 2015) for global deaths is delayed, due mainly to the reduced deaths from China in the MR-BRT model which weaken China's contribution to the global trends. The decomposition results also consistently indicate competition between population growth (dark blue) and aging (light blue) vs. improved air quality (dark red) and health care (orange). We therefore conclude that the findings from this study are robust to these complications and to the health model used.

S7. Sensitivity calculation using another decomposition approach

Based on Southerland et al.[6], it can be derived that (omitting the *i,j,k* subscripts for clarity) the logged ratio of D_{PM} between two adjacent years is:

$$log\frac{D_{PM}^{yr+1}}{D_{PM}^{yr}} = log\frac{P^{yr+1}}{P^{yr}} + log\frac{AF^{yr+1}}{AF^{yr}} + log\frac{MR^{yr+1}}{MR^{yr}} + log\frac{PAF^{yr+1}}{PAF^{yr}} \qquad (S1).$$

Approximately, we can attribute the yearly differences of D_{PM} to population change as:

$$\delta D_{PM}(P) = \frac{log\frac{P^{yr+1}}{P^{yr}}}{log\frac{D_{PM}^{yr+1}}{D_{PM}^{yr}}} \times (D_{PM}^{yr+1} - D_{PM}^{yr}) \qquad (S2).$$

The attribution to the other three factors is similarly performed.

We apply this method (i.e., the Southerland method) and the one used in the Main text (i.e., the Geng method), and find that these two methods yield nearly identical results for the 204 territories and 21 yearly changes (Fig. S6). We note that quantitative comparison between the two approaches is conducted for the first time in this work that confirms their strong consistency. In this paper, we present the results using the Geng approach to avoid division by very small numbers (used in the Southerland method) that would cause occasional outliers (e.g., points located between 10^{-6}/yr and 10^{-4}/yr, corresponding to three small island territories) in Fig. S6.

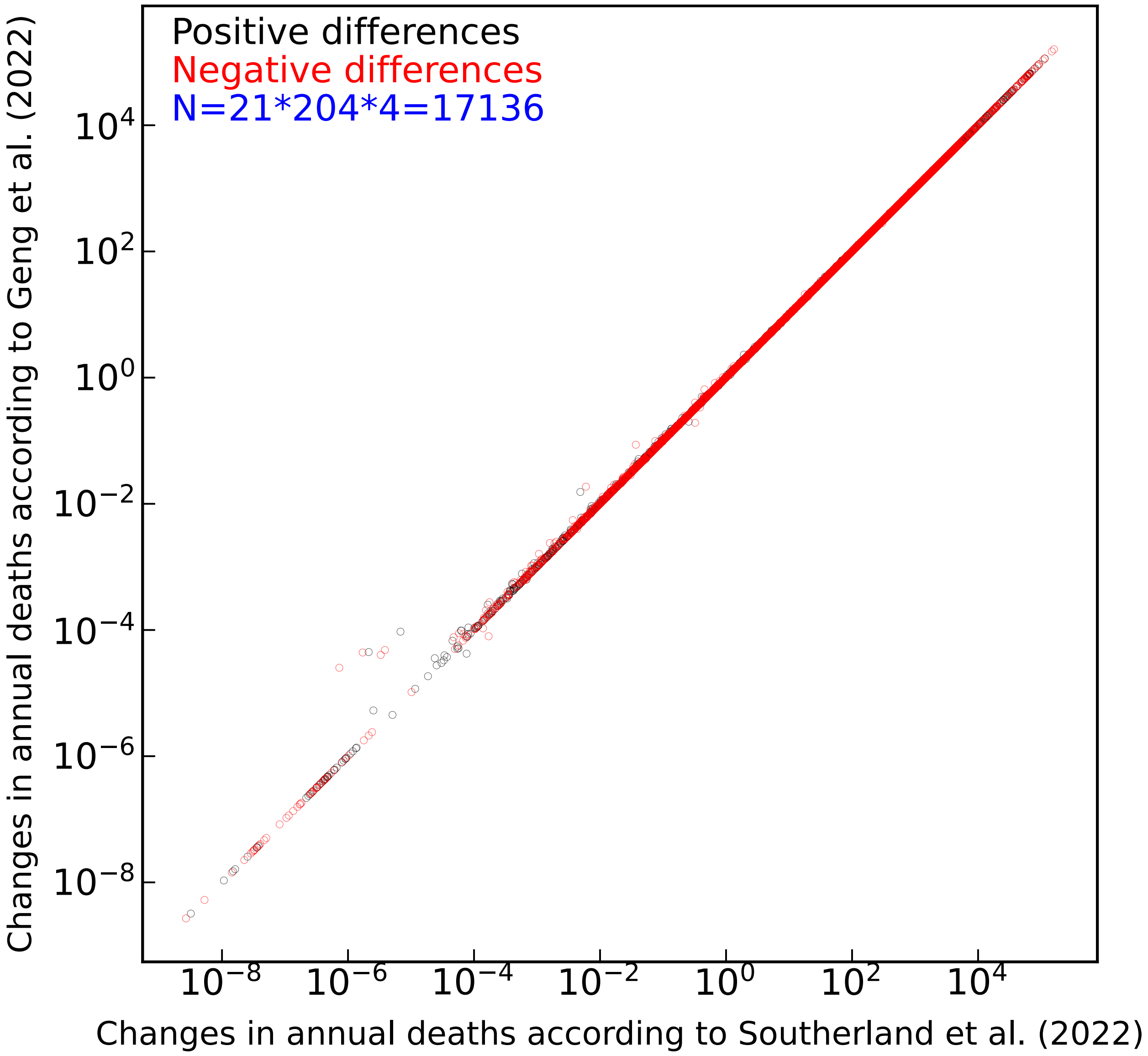

Figure S6. Comparison of attributed changes of annual $PM_{2.5}$-attributable deaths (relative to 2011) due to four factors, for 21 years and 204 territories, using the approach of (X-axis) Southerland et al.[6] and of (Y-axis) Geng et al.[8]. Negative changes are sign-reversed to accommodate the log-scale.

Supplementary References

1. van Donkelaar, A. *et al.* Monthly Global Estimates of Fine Particulate Matter and Their Uncertainty. *Environmental Science & Technology* **55**, 15287–15300 (2021).
2. Zhang, Q. *et al.* Drivers of improved PM2.5 air quality in China from 2013 to 2017. *Proceedings of the National Academy of Sciences* **116**, 24463–24469 (2019).
3. Boys, B. L. *et al.* Fifteen-Year Global Time Series of Satellite-Derived Fine Particulate Matter. *Environmental Science & Technology* **48**, 11109–11118 (2014).
4. Hammer, M. S. *et al.* Global Estimates and Long-Term Trends of Fine Particulate Matter Concentrations (1998–2018). *Environmental Science & Technology* **54**, 7879–7890 (2020).

5. Wang, H., Zhang, L., Yao, X., Cheng, I. & Dabek-Zlotorzynska, E. Identification of decadal trends and associated causes for organic and elemental carbon in PM2.5 at Canadian urban sites. *Environment International* **159**, 107031 (2022).
6. Southerland, V. A. *et al.* Global urban temporal trends in fine particulate matter (PM2·5) and attributable health burdens: estimates from global datasets. *The Lancet Planetary Health* **6**, e139–e146 (2022).
7. Singh, V., Singh, S. & Biswal, A. Exceedances and trends of particulate matter (PM(2.5)) in five Indian megacities. *Sci Total Environ* **750**, 141461 (2021).
8. Geng, G. *et al.* Drivers of PM2.5 air pollution deaths in China 2002–2017. *Nature Geoscience* **14**, 645–650 (2021).
9. NCAP, M. Ministry of Environment, Forest & Climate Change NCAP-National Clean Air Programme (2019). MoEFCC. (2019).
10. Nong, D., Wang, C. & Al-Amin, A. Q. A critical review of energy resources, policies and scientific studies towards a cleaner and more sustainable economy in Vietnam. *Renewable and Sustainable Energy Reviews* **134**, 110117 (2020).
11. Kurokawa, J. & Ohara, T. Long-term historical trends in air pollutant emissions in Asia: Regional Emission inventory in ASia (REAS) version 3. *Atmospheric Chemistry and Physics* **20**, 12761–12793 (2020).
12. Bae, M., Kim, B.-U., Kim, H. C., Kim, J. & Kim, S. Role of emissions and meteorology in the recent PM2.5 changes in China and South Korea from 2015 to 2018. *Environmental Pollution* **270**, 116233 (2021).
13. Ito, A., Wakamatsu, S., Morikawa, T. & Kobayashi, S. 30 Years of Air Quality Trends in Japan. *Atmosphere* **12**, (2021).
14. Zhang, Q. *et al.* Transboundary health impacts of transported global air pollution and international trade. *Nature* **543**, 705–709 (2017).
15. McDuffie, E. E. *et al.* Source sector and fuel contributions to ambient PM2.5 and attributable mortality across multiple spatial scales. *Nature Communications* **12**, 3594 (2021).
16. Lelieveld, J., Evans, J. S., Fnais, M., Giannadaki, D. & Pozzer, A. The contribution of outdoor air pollution sources to premature mortality on a global scale. *Nature* **525**, 367–371 (2015).
17. Che, H. *et al.* Large contribution of meteorological factors to inter-decadal changes in regional aerosol optical depth. *Atmospheric Chemistry and Physics* **19**, 10497–10523 (2019).
18. Klingmüller, K., Pozzer, A., Metzger, S., Stenchikov, G. L. & Lelieveld, J. Aerosol optical depth trend over the Middle East. *Atmospheric Chemistry and Physics* **16**, 5063–5073 (2016).
19. Zubkova, M., Boschetti, L., Abatzoglou, J. T. & Giglio, L. Changes in Fire Activity in Africa from 2002 to 2016 and Their Potential Drivers. *Geophysical Research Letters* **46**, 7643–7653 (2019).
20. Muthoni, F. K. *et al.* Long-term spatial-temporal trends and variability of rainfall over Eastern and Southern Africa. *Theoretical and Applied Climatology* **137**, 1869–1882 (2019).
21. Reddington, C. L. *et al.* Air quality and human health improvements from reductions in deforestation-related fire in Brazil. *Nature Geoscience* **8**, 768–771 (2015).
22. Aragão, L. E. O. C. *et al.* 21st Century drought-related fires counteract the decline of Amazon deforestation carbon emissions. *Nature Communications* **9**, 536 (2018).
23. Guo, J. *et al.* A merged continental planetary boundary layer height dataset based on high-resolution radiosonde measurements, ERA5 reanalysis, and GLDAS. *Earth System Science Data Discussions* **2022**, 1–33 (2022).
24. Huang, X. *et al.* Chemical Boundary Layer and Its Impact on Air Pollution in Northern China. *Environmental Science & Technology Letters* **7**, 826–832 (2020).
25. Cheng, Y. *et al.* Reactive nitrogen chemistry in aerosol water as a source of sulfate during haze events in China. *Science Advances* **2**, e1601530 (2016).
26. Gunthe, S. S. *et al.* Enhanced aerosol particle growth sustained by high continental chlorine emission in India. *Nature Geoscience* **14**, 77–84 (2021).
27. Yun, X. *et al.* Residential solid fuel emissions contribute significantly to air pollution and associated health impacts in China. *Science Advances* **6**, eaba7621 (2020).
28. Cohen, A. J. *et al.* Estimates and 25-year trends of the global burden of disease attributable to ambient air pollution: an analysis of data from the Global Burden of Diseases Study 2015. *The Lancet* **389**, 1907–1918 (2017).
29. Bloom, D. E. *et al.* Macroeconomic implications of population ageing and selected policy responses. *The Lancet* **385**, 649–657 (2015).
30. Lutz, W., Sanderson, W. & Scherbov, S. The coming acceleration of global population ageing. *Nature* **451**, 716–719 (2008).

31. Murray, C. J. L. *et al.* Global burden of 87 risk factors in 204 countries and territories, 1990–2019: a systematic analysis for the Global Burden of Disease Study 2019. *The Lancet* **396**, 1223–1249 (2020).
32. Burnett, R. *et al.* Global estimates of mortality associated with long-term exposure to outdoor fine particulate matter. *Proceedings of the National Academy of Sciences* **115**, 9592–9597 (2018).
33. Tao, S. *et al.* Quantifying the rural residential energy transition in China from 1992 to 2012 through a representative national survey. *Nature Energy* **3**, 567–573 (2018).
